# Supplementary material for: Spatial clustering and temporal trend analysis of international migrants diagnosed with tuberculosis in Brazil
Source: PLoS One. 2021 Jun 9;16(6):e0252712. doi: 10.1371/journal.pone.0252712 (PMC8189475; doi:10.1371/journal.pone.0252712)
Supplement: Attachment — Submitted filename: Response to Reviewers.docx [file pone.0252712.s001.docx]

**Dear Editor,**

Many thanks for your reply and your reviewers' comments about our manuscript **“PONE-D-20-40788 - Spatial clustering and temporal trend analysis of international migrants diagnosed with tuberculosis in Brazil”**.

The comments were appropriate to qualify/improve the manuscript. We have forwarded a letter with the changes made in the manuscript, presenting our response for each comment from reviewers, as requested. Many thanks for the comments.

Kind regards,

Arcêncio et al.

**Editor comments:**

**Comment:** 1. Thank you for stating in your Funding Statement:
 [NO - he funders had no role in study design, data collection and analysis, decision to publish, or preparation of the manuscript.].

- Please provide an amended statement that declares *all* the funding or sources of support (whether external or internal to your organization) received during this study, as detailed online in our guide for authors at <http://journals.plos.org/plosone/s/submit-now>.  Please also include the statement “There was no additional external funding received for this study.” in your updated Funding Statement.

* Please include your amended Funding Statement within your cover letter. We will change the online submission form on your behalf.

**Response:** *Thank you for your comment. We inform that the author ELMN received received funding from the Pan American Health Organization (PAHO) [award number: 67278]. According to the journal's submission rules, this information was not included in the manuscript, only in the form of the submission system. We also inserted this statement in our cover letter, as directed.*

**Comment:** 2. Please amend your authorship list in your manuscript file to include author Tatiana Ferraz de Araújo Alecrim

**Response:** *Thank you for your observation and sorry for the mistake. This has already been corrected in the manuscript.*
