# Supplementary material for: Spatial clustering and temporal trend analysis of international migrants diagnosed with tuberculosis in Brazil
Source: PLoS One. 2021 Jun 9;16(6):e0252712. doi: 10.1371/journal.pone.0252712 (PMC8189475; doi:10.1371/journal.pone.0252712)
Supplement: Attachment — Submitted filename: Response to Reviewers.docx [file pone.0252712.s002.docx]

**Dear Editor,**

Many thanks for your reply and your reviewers' comments about our manuscript **“PONE-D-20-40788 - Spatial clustering and temporal trend analysis of international migrants diagnosed with tuberculosis in Brazil”**.

The comments were appropriate to qualify/improve the manuscript. We have forwarded a letter with the changes made in the manuscript, presenting our response for each comment from reviewers, as requested. Many thanks for the comments.

Kind regards,

Arcêncio et al.

**Editor comments:**

**Comment:** Please review your reference list to ensure that it is complete and correct. If you have cited papers that have been retracted, please include the rationale for doing so in the manuscript text, or remove these references and replace them with relevant current references. Any changes to the reference list should be mentioned in the rebuttal letter that accompanies your revised manuscript. If you need to cite a retracted article, indicate the article’s retracted status in the References list and also include a citation and full reference for the retraction notice.

**Response:** *Thank you for your comment. All references have been revised.*

**Reviewer #2:**

**Comment:** Abstract: Please revise the sentence “The study revealed the impact of TB in migrants. Based on the findings, if TB surveillance is not intensified in these areas, there will likely be an increase in cases in adjacent regions, making the target defined by the End TB Strategy more difficult to achieve”. How can the paper really revealed the impact of TB in migrants if it is not possible to determine the incidence of TB among migrant populations? If TB cases are being diagnosed and treated, why would the authors expect an increase in cases in adjacent regions is expected?

**Response:** *Thank you for your comment and we agree with your observation so that we rewrite the conclusion of the abstract.*

**Comment:** Introduction: The sentence “Several studies have shown the impact of migration on the resurgence and spread of TB in Europe” is inaccurate. The references 2 and 6-11 do show that cases among migrants pose challenges to local programmes but not necessarily that transmission contributes to it – e.g., Hargreaves (2017) show that there is low proportion of cases due to transmission from migrants to the local community, and that this is restricted to some communities. I suggest to remove the word spread.

**Response:** *Thank you for your comment, we agree to observe it and as suggested, we remove the word "spread" from the sentence.*

**Comment:** Discussion: Page 18, row 338 – It needs to be more clear that the authors are referring to cases in “temporal trends of TB in migrants as increasing”.

**Response:** *Thank you for your comment. We rewrote the sentence to make it clearer, as suggested.*

**Comment:** Page 21, rows 387-393 - The authors say that TB cases among migrants in São Paulo have been stationary since 2014 and “Another hypothesis is underreporting and/or underdiagnosis in this population from 2014 to 2017, since the city did not report any migrants diagnosed with TB during that period.”. Nevertheless, the study by Pescarini (2018) (ref 38) reports data on over 335 migrants from South-American countries in 2013/2014 in Central areas from the municipality of São Paulo. There was any change in the National or State TB registry that may have caused this change? Please clarify the differences found and how these changes could have impacted the analysis.

**Response:** *This is an excellent observation that also made us thoughtful.*

*The data from our study were obtained through SINAN, which is the official notification system in Brazil, as well as data from the study by Pescarini (2018), which also used data provided by the laboratories of the Adolfo Lutz Institute (IAL).*

*The SINAN database should be the same, both for us and for Pescarini. We were unable to say about the database received by Pescarini, but in our databank there are approximately 45 thousand cases of TB reported in São Paulo only during the period under study (2014 to 2019), with 7 thousand cases only in 2014, but not there were TB cases registered as being in migrants between the years 2014 and 2017 in São Paulo. It is worth mentioning that in Pescarini's study, he selected only the central region of São Paulo and here we are talking about the municipality as a whole, so the numbers I quote here are different from the numbers presented in Pescarini's study.*

*Considering that theoretically the SINAN bank is the same for everyone who has access, we can conclude that the cases of migrants considered by Pescarini in his study were obtained through the IAL laboratory exam database, so that when the exam was carried out , the person reported being a migrant, but that information was not entered in SINAN. Knowing these people, it is possible to cross-check the databases to obtain the other variables that Pescarini used in his study.*

*Studies using secondary databases are subject to these limitations regarding unreported data, typing errors and other issues, as discussed in the manuscript, so that the analyzes made and presented were carried out with methodological rigor in order to reduce possible biases, however we work with the data provided to us and we are able to answer only for them.*

*Sorry if we could not clarify your question with further information. We inform that we inserted a paragraph in the discussion session referring to the possibility of crossing databases from different sources to minimize the bias already mentioned.*
